# Thermal Stability of Organic Semiconductor Thin Film Glasses by Local Changes in Spontaneous Orientation Polarization

**DOI:** 10.1021/acs.jpcb.5c01679

**Published:** 2025-04-07

**Authors:** M. Ruiz-Ruiz, A. Villalobos-Martin, T. Bar, C. Rodriguez-Tinoco, J. Fraxedas, S. Capaccioli, M. Labardi, M. Gonzalez-Silveira, J. Rodriguez-Viejo

**Affiliations:** † Departamento de Física. Facultad de Ciencias, 16719 Universitat Autònoma de Barcelona, Bellaterra 08193, Spain; ‡ 231882 Catalan Institute of Nanoscience and Nanotechnology (ICN2), CSIC and BIST, Campus UAB, Bellaterra 08193, Barcelona, Spain; § Institute for Chemical and Physical Processes (IPCF), National Research Council (CNR), Pisa Research Area, Via Moruzzi 1, Pisa 56124, Italy; ∥ Department of Physics “Enrico Fermi”, University of Pisa, Largo Pontecorvo 3, Pisa 56127, Italy

## Abstract

Vapor-deposited organic semiconductor glasses exhibit distinct molecular anisotropy and exceptional kinetic and thermodynamic stability, distinguishing them from the inherently isotropic and poorly stable glasses formed through liquid cooling. In this study, we exploit these unique properties to examine local changes in surface potential as the stable glass transitions to a supercooled liquid upon heating above the glass transition temperature (*T*_g_). Vapor deposited glasses of organic molecules with permanent dipole moments can generate a measurable surface potential due to their anisotropic molecular orientation. We use local electrostatic force microscopy and Kelvin probe force microscopy to provide insights into the dynamics of the phase transformation occurring above *T*_g_. We demonstrate that changes in polarization upon conversion to the isotropic liquid serve as an effective proxy for tracking this transition and highlight their potential for evaluating the thermal stability of organic devices under diverse thermal conditions.

## Graphical abstract

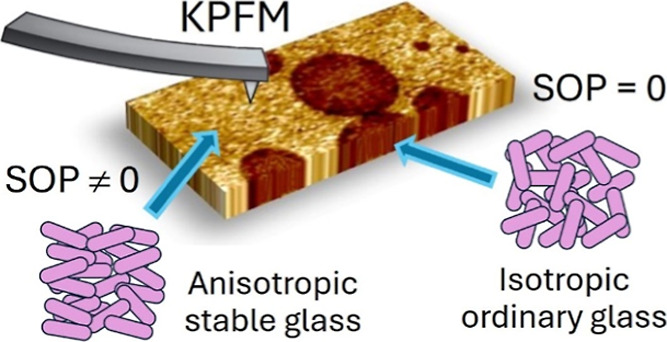

## Introduction

Ultrastable glasses (UG), prepared by vapor deposition at specific temperatures, exhibit higher density compared to liquid-cooled glasses (LCG), with molecules arranged in tightly packed environments characterized by very low mobility and significantly extended relaxation times.^1,2^ During heating, thin film UGs transform into a supercooled liquid (SCL) through a mechanism distinct from that of conventional, liquid-cooled glasses. Only molecules near free surfaces or weakly interacting interfaces possess sufficient mobility to trigger the transformation into the SCL. Thus, in supported films with a free surface, heating the sample above the glass transition temperature (*T*_g_) initiates the transformation at the free surface, progressing through a growth front that consumes the remaining sample.^3–5^ Once the liquid forms at the free surface, kinetic facilitation drives the consumption of the remaining glass at a constant velocity under isothermal conditions.^6^ This surface-driven mechanism can be inhibited in thin films by applying capping layers of a higher *T*_g_ material on both the top and bottom of the low *T*_g_ middle layer, forming a trilayer structure.^7,8^ This approach has been previously employed to block the growth front, enabling a bulk-like transformation. Inhibiting surface mobility results in a highly heterogeneous transformation into the liquid state with time scales on the order of τ ∼ 10^6^ to 10^7^τ_α_,^9–12^ with τ_α_ = 100 s at *T*_*g*._. For instance, if a glass formed by cooling a liquid at a rate of −10 K/min (referred to as an ordinary glass) transforms in 100 s when heated at its *T*_g_, an ultrastable glass would take approximately 10^9^ seconds (or 30 years) to transform at the same temperature, and presumably much longer below it.

This regime has been experimentally observed using nanocalorimetry^10,11^ and, more recently, atomic force microscopy (AFM).^13^ Relaxation maps of the liquid formation were constructed during isothermal measurements above the glass transition temperature *T*_g_ as a function of time. These maps are based on the impact of the liquid phase of the intermediate layer on the topography of the trilayers, attributed to compressive stresses arising from the differing thermal expansion coefficients of the organic glasses and the silicon substrate. When a specific region of the material transforms into liquid, the compressive stress induces buckling in the layers, and surface corrugation becomes detectable by AFM. As the liquid regions expand laterally, the surface exhibits characteristic wrinkling with peaks and valleys and specific wavelengths. Since it is extremely challenging to structurally distinguish a glass and its supercooled liquid at local levels, this methodology provided a convenient way to map the formation of the liquid. However, it only provides indirect evidence of liquid formation through the impact on the mechanical properties and the appearance of surface corrugation.^13,14^

An intriguing characteristic of vapor-deposited glasses is their tendency to exhibit molecular anisotropy, where molecules are generally aligned in a specific direction—either in-plane, out-of-plane, or isotropically. This alignment depends on factors such as the shape anisotropy of the individual molecules, the deposition temperature, the deposition rate,^15–19^ or light illumination during growth.^20^ This contrasts with liquid-cooled glasses, which are isotropic, and presents interesting opportunities to introduce an additional degree of freedom to the properties of vapor-deposited glasses. For instance, appropriate molecular packing that favors π–π interactions may enhance electronic transport.^21^ The difference in molecular orientation between highly stable vapor-deposited glasses and liquid-cooled glasses also provides an additional proxy to monitor the transformation of anisotropic glasses into the supercooled liquid upon heating above *T*_g_. This phenomenon has already been demonstrated using grazing-incidence wide-angle X-ray scattering (GIWAXS).^22–24^

If individual molecules possess a permanent dipole moment (PDM), even a small one, molecular orientation can lead to notable average polarization and voltage drops across vapor-deposited thin films.^25,26^ This phenomenon typically occurs perpendicular to the substrate due to growth constraints. When the average PDM orientation is maintained throughout the film thickness, the surface potential increases linearly without reaching saturation. The accumulation of a substantial surface potential in vapor-deposited thin film glasses, known as spontaneous orientation polarization (SOP), can significantly impact device performance. A buildup of surface potential was first reported in 1972 upon gas condensation on cold substrates.^27^ However, SOP is generating renewed interest within the organic electronic community due to its beneficial or detrimental impacts on the behavior of organic devices.^18,26,28–30^ For example, SOP can enhance charge injections, thereby lowering the voltage threshold for the on-state of an OLED.^25,31^ Conversely, polarization can induce surface charges at the interfaces between polar and nonpolar materials, reducing the power conversion efficiency of OLEDs due to increased exciton-polaron quenching.^29^ Recently, He et al. applied this SOP formalism to optimize OLED devices, maximizing their efficiency.^30^ Molecular orientation also influences energy harvesting devices. Organic glassy layers of TPBi or Alq_3_ with SOP, forming a plate-capacitor device, have been shown to generate electric currents under mechanical oscillation.^32^

While the presence of PDM in individual molecules is necessary for SOP, the reverse is not true. There is no clear correlation between the permanent dipole moment of molecules and the giant surface potential (GSP) of films; molecules with large PDM may result in films with low polarization. This is likely due to dipole–dipole interactions, which tend to produce antiparallel orientation of neighboring molecules during growth, e.g., dimerization.^33,34^ SOP has been measured in a variety of organic semiconductors with values up to around 160 mV/nm depending on the specific molecule.^33,35^ Negative polarization can also be achieved in some cases.^33^ The significant difference in SOP between liquid-cooled glasses (isotropic and thus exhibiting very small SOP) and vapor-deposited glasses (anisotropic and potentially exhibiting larger SOP) provides a novel method to assess the thermal stability of thin film organic semiconductor glasses. This distinction is particularly useful for locally mapping the transformation of vapor-deposited glasses into a supercooled liquid. The comparison becomes even more impactful if the formation of the supercooled liquid proceeds through a highly heterogeneous process, as recently demonstrated in capped ultrastable thin-film glasses.^13^ In such cases, submicron patches of liquid emerge and grow through dynamic facilitation, consuming the stable glass. However, direct identification of the liquid state with in-plane spatial resolution has remained elusive. To date, the liquid nature of these regions has been inferred only indirectly, either through (nano)­calorimetry^5,10^ or, more recently, by analyzing their influence on the wrinkling of the rigid top surface layer.^13^ Notably, organic semiconductor glasses formed around 0.85 *T*_g_ exhibit molecular anisotropy^36^ and surface polarization.^18^ This observation suggests that techniques sensitive to dipolar orientation and surface potential buildup could enable in situ local mapping of its formation during thermal treatments. Electrical-based AFM methodologies, offering exceptional spatial resolution and high sensitivity to electrical variations in the near-surface region, are particularly well-suited for this purpose.^37,38^

Here, we introduce an electrical-based AFM approach for the direct identification of supercooled liquid regions emerging during the heterogeneous transformation of an ultrastable glass, as well as liquid-cooled glass domains formed upon returning to room temperature. This method measures variations in polarization resulting from changes in molecular orientation. We demonstrate that this approach can be used to monitor the slow dynamics of the transformation at temperatures just a few K above the glass transition temperature of the ordinary glass. Our results show a good correlation between topography and electrical-based images, although we were able to assess that the liquid-cooled glass extends slightly beyond the region of mechanical deformation. This new methodology provides enhanced resolution in identifying the interface between the liquid-cooled and ultrastable glass, allowing for precise tracking of its evolution over time, starting from the initial stages of formation, when mechanical deformations could not yet arise.

## Materials and Methods

### Vapor Deposition of Thin Film Trilayers

Trilayer samples of tris­(4-carbazoyl-9-ylphenyl)­amine (TCTA)/(*N*,*N*′-bis­(3-methylphenyl)-*N*,*N*′-diphenylbenzidinem (TPD)/TCTA are grown in a high vacuum chamber with a low pressure around 10^–8^ mbar by vapor deposition at a deposition temperature of *T*_dep_ = 285 K, which corresponds to 0.85 *T*_g_ of the ordinary liquid-cooled TPD glass, i.e. *T*_g_ (10 K/min) = 333 K.^8,39^ TPD and TCTA with purity 99% were obtained from Sigma-Aldrich and used as received. The growth rate monitored through a quartz crystal monitor was 0.085 ± 0.015 nm/s for all layers. The analyzed samples consist of layers with thicknesses of 13, 65, and 13 nm, deposited on the native oxide layer of a boron-doped (p-type) Si(001) substrate. The silicon wafer used for the substrate has a resistivity ranging from 0.001 to 0.005 Ω·cm and a thickness of 525 ± 25 μm. The whole process is described in more detail in previous publications.^8,13^ Some of the samples are transformed ex situ at temperatures 12–16 K above *T*_g_ to initiate the transformation and then measured by AFM, while others are thermally treated in situ during AFM operation in dry N_2_ atmosphere in a sealed chamber implemented to the AFM measurement head (see below).

### AFM-Based Methodologies

An AFM from Veeco Instruments Ltd. (Sunnyvale, CA, USA), model MultiMode, with controller Nanoscope IIIa, Quadrex and ADC5 extension, has been used for the electrostatic force microscopy (EFM) and Kelvin Probe Force Microscopy (KPFM) measurements. To such purpose, the microscope was adapted to work in frequency modulation (FM) mode by the addition of a phase-locked loop (PLL) controller (model PLLProII, RHK Technology, Troy, MI, USA), a couple of dual-channel lock-in amplifiers (model SR830DSP, Stanford Research Systems, Sunnyvale, CA, USA), and some home-built electronics. The microscope is equipped with a sealed gas cell for measurements in controlled atmosphere and a thermal application controller (TAC, Veeco Instruments) for stabilization of the sample temperature. All measurements were conducted at *T* = 37 °C and in dry nitrogen atmosphere, unless specified. Working in dry conditions is mandatory in order to strongly reduce the influence of highly polarizable water molecules.^40^ Platinum-coated AFM silicon cantilevers (HQ:DPER-XSC11 type C, MikroMasch, Sofia, Bulgaria) were used for allowing electrical modes, with apical tip radius of about 20 nm, spring constant *k* ∼ 7 N/m, resonant frequency *f*_0_ ∼ 168 kHz, and quality factor of *Q* ∼ 350.

All measurements were made in the AFM intermittent contact mode, with an oscillation amplitude *a* of the cantilever stabilized by topography feedback to around 15 nm, with a ratio to the free amplitude *a*_0_ of *a*/*a*_0_ ∼ 80–90%, that resulted as the most convenient one for the evaluation of dielectric properties of our thin films.^41^ EFM is performed in the frequency-modulated mode, by application of an external electric potential *V*(*t*) = *V*_dc_ + *V*_ac_ cos­(Ω*t*) to the conductive tip, and detection of the related interaction force gradient, that allows improved resolution compared to the detection of bare forces.^37^ In this mode, a resolution of 3 nm was demonstrated on polymer multilayer samples,^42^ while resolution better than 5 nm was assessed on self-assembled block copolymer lamellar nanostructures.^41^

The system made up of the conductive tip faced to the sample surface is characterized by the capacitance *C*(*z*), where *z* is the tip/surface distance. By application of *V*(*t*), an electric force *F*_el_(*t*) arises, whose gradient amounts to

1 $$\frac{dF_{el}}{dz}\left(t\right)=\frac{1}{2}V^{2}\left(t\right)\frac{d^{2}C}{dz^{2}}=\frac{dF_{c}}{dz}+\frac{dF_{\Omega }}{dz}\cos\left(\Omega t\right)+\frac{dF_{2\Omega }}{dz}\cos\left(2\Omega t\right)$$

with

2 $$\frac{dF_{dc}}{dz}=\frac{1}{2}\left(V_{dc}^{2}+\frac{1}{2}V_{ac}^{2}\right)\frac{d^{2}C}{dz^{2}}$$

3 $$\frac{dF_{\Omega }}{dz}=2V_{dc}V_{ac}\frac{d^{2}C}{dz^{2}}$$

4 $$\frac{dF_{2\Omega }}{dz}=\frac{1}{4}V_{ac}^{2}\frac{d^{2}C}{dz^{2}}$$

In the frequency modulation mode, the instantaneous resonance frequency shift of the cantilever, Δ*f*_res_(*t*) = *f*_res_(*t*) – *f*_0_, is proportional to d*F*_el_/d*z*. The signal components can be measured separately by lock-in demodulation of Δ*f*_res_(*t*) both at frequency Ω and 2Ω. Notably, the second-harmonic component, Δ*f*_2Ω_, only contains information about the system capacitance, that can be used to derive the dielectric permittivity of the surface by proper modeling.^43^ The component Δ*f*_Ω_, instead, can be used to detect the amount of surface static charge, as well as the contact potential difference, *V*_CPD_, between the tip material (Pt in our case) and the facing surface portion of the sample. This last kind of measurement, named KPFM, is implemented by using a nulling method, since the term of eq 3 can be nulled when *V*_dc_ goes to zero. In the case of a built-in surface potential, *V*_dc_ in eqs 1–3, should be replaced by the term (*V*_dc_ + *V*_CPD_). Therefore, by measuring Δ*f*_Ω_, an additional feedback loop can be used to adjust the value of *V*_dc_ until Δ*f*_Ω_ is brought to zero. The necessary *V*_dc_ value thus equates – *V*_CPD_.

Performance of KPFM in the frequency modulation mode allows to greatly improve its spatial resolution, down to the level of a few nm.^38^ Both EFM and KPFM methods performed in frequency-modulation mode as in our case were recently reviewed.^44^

### UV/Vis Spectroscopy

Measurements were carried out in a Shimadzu UV1280 UV/vis Spectrophotometer equipped with a custom-made gas cell to heat the sample above *T*_g_ in an inert atmosphere. TPD exhibits two primary electronic transitions in its UV–vis spectra, observed at 355 nm (3.50 eV) and 315 nm (3.95 eV), respectively, as illustrated in Figure 1. The lowest energy transition corresponds to the HOMO–LUMO transition. In this transition, the HOMO state of the molecule is distributed across the entire molecule, with contributions from the amine groups, the biphenyl core, and the peripheral C rings. The LUMO state, in contrast, is predominantly localized on the biphenyl core, suggesting that the transition dipole moment (TDM) of the molecule aligns along its long axis. The higher energy transition, on the other hand, involves two nearly degenerate transitions, that are localized on the peripheral C rings. This transition is associated with two distinct transition dipole moments, oriented in the plane perpendicular to the long axis and opposite directions.^45^ The intensity of the transition at 355 nm (3.50 eV) is used as a reference to assess the molecular orientation in comparison to an isotropic sample.

**Figure 1:**
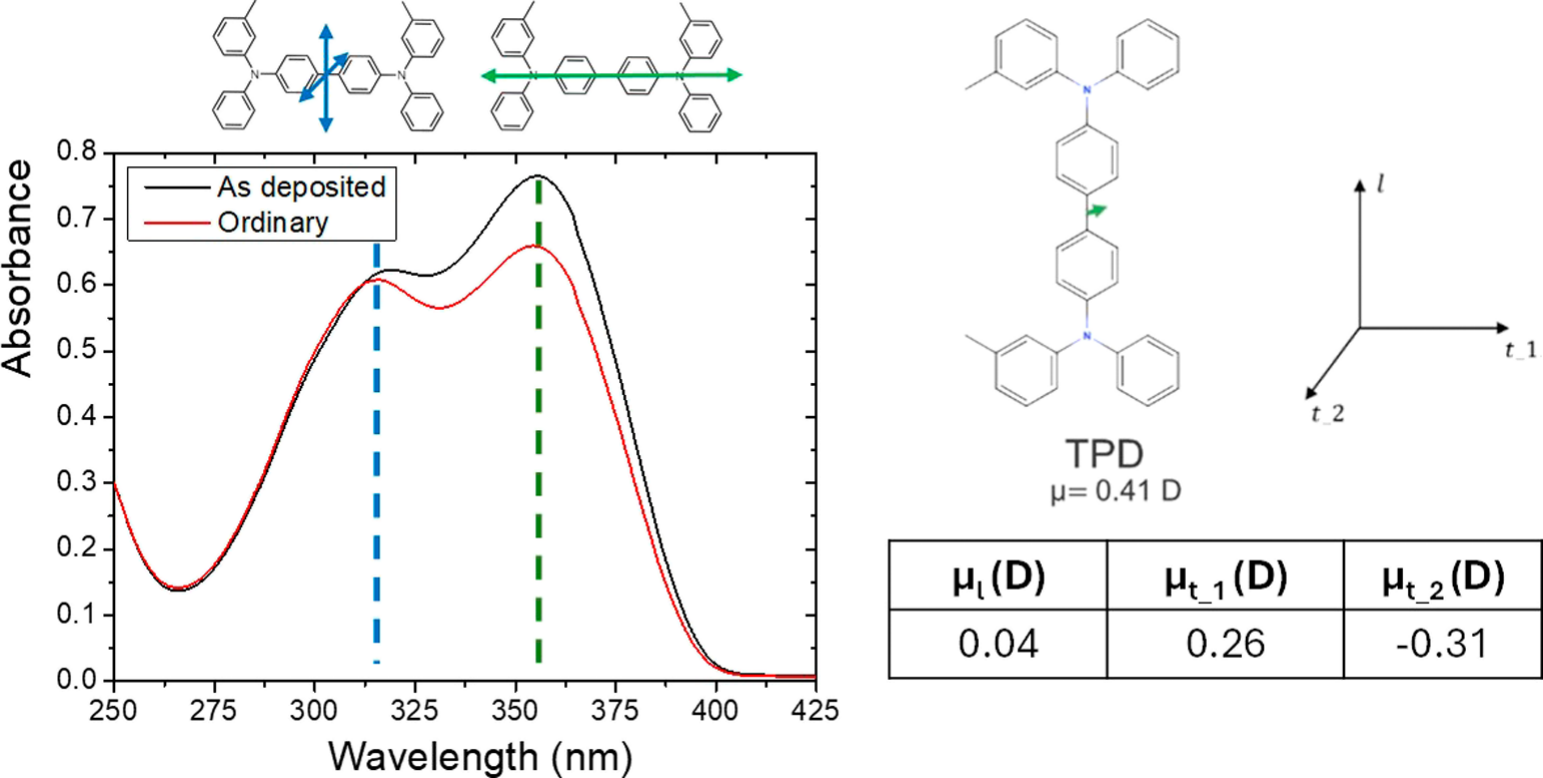
UV/vis absorbance spectra of a 100 nm thick TPD film (black line) deposited at 0.85 *T*_g_, and of the same film after being annealed above *T*_g_ until it completely transformed into a liquid and cooled down to RT (red line). At the top, the chemical structure of the molecule is reported, with the TDMs indicated in cyan/green on the left. The electric dipole moment vector is shown in green in the right image. Numerical values correspond to the coordinates and modulus of the electric dipole moment of TPD with respect to the shown axes. The electric dipole moment of the single TPD molecule was obtained by first-principles calculations in a previous publication.^46^

The absorbance of this transition relates to the extent of horizontal orientation of the molecules relative to the substrate. The degree of molecular orientation is quantified using the order parameter, *S*_*z*_, which is typically derived from the angle θ between the transition dipole moment and the surface normal, but it can be simplified to a ratio of absorbances^47^

5 $$S_{z}=\frac{1}{2}\left(3\left\langle \cos^{2}\theta \right\rangle -1\right)\to S_{z}=1-\frac{A}{A_{iso}}$$

Here, *A* represents the absorbance of the as-deposited film, while *A*_iso_ is the absorbance of the same film but with random molecular orientation. This isotropic condition is achieved by annealing the vapor-deposited film for 5 min at 347 K (*T*_g_ + 14 K) in an Ar atmosphere. Then, the film is passively cooled down to room temperature at an estimated rate of −5 K/min across the glass transition region. An *S*_*z*_ value of −0.5 indicates that the TDM aligns parallel to the substrate, reflecting a horizontal molecular orientation; *S*_*z*_ = 0 means random orientation of the molecules, while *S*_*z*_ = 1 indicates that the molecules are preferentially aligned with their long axis perpendicular to the substrate. Although absorbance spectra are recorded for single TPD layers, and EFM is instead conducted on trilayers, we do not expect variations of the molecular orientation in the intermediate TPD film due to the underlying TCTA layer.^8,11^ Indeed, it has been established that anisotropic packing in vapor-deposited organic glasses is mainly driven by surface equilibration mechanisms during the growth process.^48^

## Results and Discussion

As recently visualized by AFM topography,^13^ the transformation of the capped ultrastable thin film glass into the supercooled liquid above *T*_g_ occurs through a highly heterogeneous process, where liquid-like regions emerge and grow, consuming the highly stable glassy matrix. Upon cooling, these regions transform into liquid-cooled glass, while the nontransformed regions retain the characteristics of a stable glass.^10,11^ If the annealing process is stopped before the transformation is complete, the sample consists of differentiated regions of two distinct glasses characterized by markedly different molecular ordering, stabilities, and dynamics. The regions of stable glass that have not yet transformed into the liquid phase are expected to retain significant molecular anisotropy. In the case of TPD, this anisotropy is anticipated to generate a measurable buildup of surface potential. In contrast, the liquid-cooled glassy regions, characterized by isotropic molecular orientation, are predicted to exhibit negligible net polarization across their thickness.

Figure 2 demonstrates the capability of electrical-based AFM to locally differentiate between liquid-cooled glass (LCG) and ultrastable glass (UG) based on their surface potential variations. The imaged sample is a trilayer that underwent thermal treatment at *T*_g_ + 16 K for 90 min. Figure 2a presents a topography map showing a broad distribution of wrinkled regions associated with the LCG, formed during the thermal treatments above *T*_g_ and subsequent cooling to room temperature. Figure 2b displays the Δ*f*_Ω_ signal, which is sensitive to surface charges and thus proportional to the buildup of surface potential due to anisotropic PDM orientation within the thin film glass. This results in an electrical contrast arising from the small potential difference between the two types of glasses. Notably, the distribution observed in Figure 2b closely matches the topographic images (Figure 2a), with the LCG domains extending slightly beyond the topographic undulations. Figure 2c provides a scanning map in KPFM mode, measuring the *V*_CPD_ with respect to the Platinum tip, offering a quantitative measure of the surface potential differences between the regions. Darker colors indicate lower surface potential (in absolute terms), clearly showing the higher surface potential of the UG compared to the LCG. Figure 2d shows the Δ*f*_2Ω_ signal, which is related to the dielectric constant, appearing as quite homogeneous, as the features observed in this image are mainly due to topographic variations, affecting the tip/surface capacitance.

**Figure 2:**
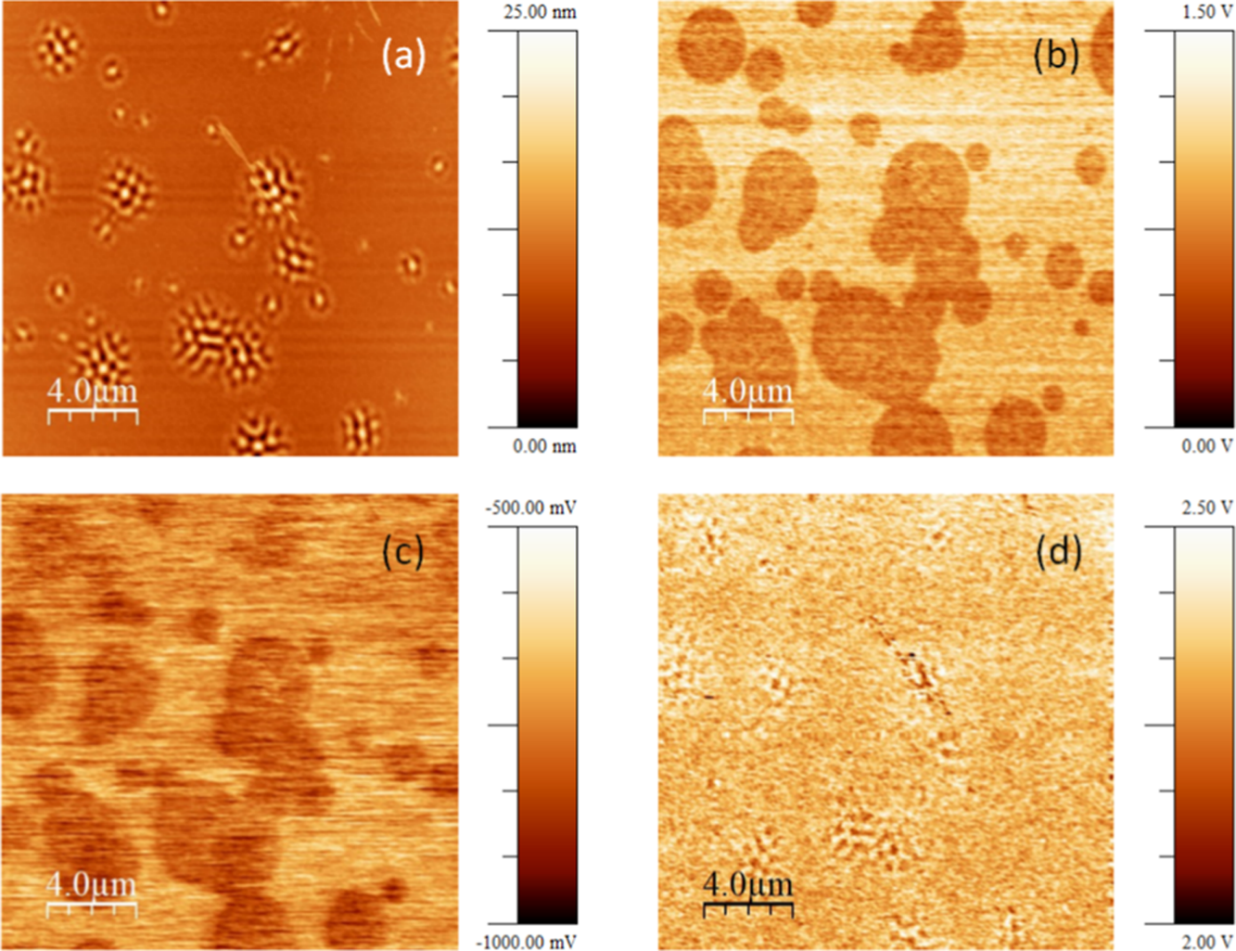
Room temperature AFM images of the same region (20 μm × 20 μm) of a partially transformed trilayer sample in different scanning modes: (a) topography; (b) Δ*f*_Ω_ (in arbitrary units); (c) *V*_CPD_ with respect to platinum, measured by KPFM; (d) Δ*f*_2Ω_ (in arbitrary units).

Molecular orientation was assessed using UV/vis spectrophotometry on single-layer TPD films, each 100 nm thick, deposited on sapphire substrates. The analysis was performed by examining their absorbance spectra. Comparisons were made between glasses vapor-deposited at 285 K (corresponding to 0.85 *T*_g_ for TPD), and their liquid-cooled glass counterparts, obtained after heating above *T*_g_ and cooling back to room temperature (see Materials and Methods for details). The UV/vis spectra of Figure 1 show a change in the absorbance of the transition at 355 nm which indicates the long axis of the molecule has a net orientation compared to the LCG. According to Figure 1 and eq 5, *S*_*z*_(UG) = −0.14 ± 0.02, taking the LCG as the isotropic state (*S*_*z*_ = 0). This calculation yields θ = 61 ± 1°, indicating that the long axis of the molecule forms on average a 61° angle with the surface normal. This value suggests that, on average, the molecules are aligned in a predominantly face-on orientation, with a tendency to lie parallel to the substrate surface. The negative value of *S*_*z*_ aligns with previous findings toward a slight horizontal orientation growing at 0.85 *T*_g_,^36^ but is slightly larger than our earlier measurements.^49^ Figure 1 (top) also sketches the TPD molecule, with the TDMs along and perpendicular to the long axis (left), and its permanent dipole moment (PDM) (right) with components outlined also in Figure 1. The different surface potential observed in Figure 2 within the UG region, attributed to the orientation of the dipole moment, also suggests a slight rotation of the molecules around their long axis (as discussed below).

To provide a more quantitative analysis of the surface potential differences between liquid-cooled glass (LCG) and ultrastable glass (UG) regions, we conducted high-resolution spatial scans in four modes along a line traversing from the UG matrix, through a single LCG region, and back into the UG, as shown in Figure 3a. Figure 3b represents the average of 32 consecutive scans to enhance the signal-to-noise ratio. The topography line scan (black symbols) reveals peaks and valleys in the wrinkled region associated with the LCG domains, with a maximum vertical amplitude of 25 nm and a lateral size of approximately 2 μm. The Δ*f*_Ω_ signal (red symbols) indicates a distinct step between the LCG and UG domains. The LCG domain spans approximately 2.8 μm, slightly exceeding the topographic one. This trend is consistently observed across all LCG domains, as qualitatively inferred from Figure 2a,b and shown in more detail in Figure 3c. The KPFM signal (blue symbols) mirrors the EFM signal but provides a quantitative measure of the surface potential difference between the LCG and UG domains. This difference is quantified as 150 ± 20 mV, which, assuming the surface potential of the LCG is near zero, implies a value of 2.4 ± 0.2 mV/nm for the TPD film grown at 285 K, which is comparable to the giant surface potential found for similar molecules with low PDM, such as α-NPD (PDM = 0.34 D and GSP of 5 mV/nm).^25^ We consider the contribution of the TCTA capping layer to the relative surface potential variation between LCG and UG to be negligible since the PDM of TCTA is zero^35^ and it is homogeneously distributed across the whole surface with a small thickness of 13 nm, although slight alterations of the polarization value cannot be entirely ruled out. As previously noted, the Δ*f*_2Ω_ signal (Figure 2d) is primarily sensitive to topographic variations. Furthermore, the contribution from unavoidable surface contamination upon exposure to atmosphere should be negligible since we are interested in surface potential differences, i.e., the surface is supposed to be homogeneously contaminated,^50^ and not in absolute values.

**Figure 3:**
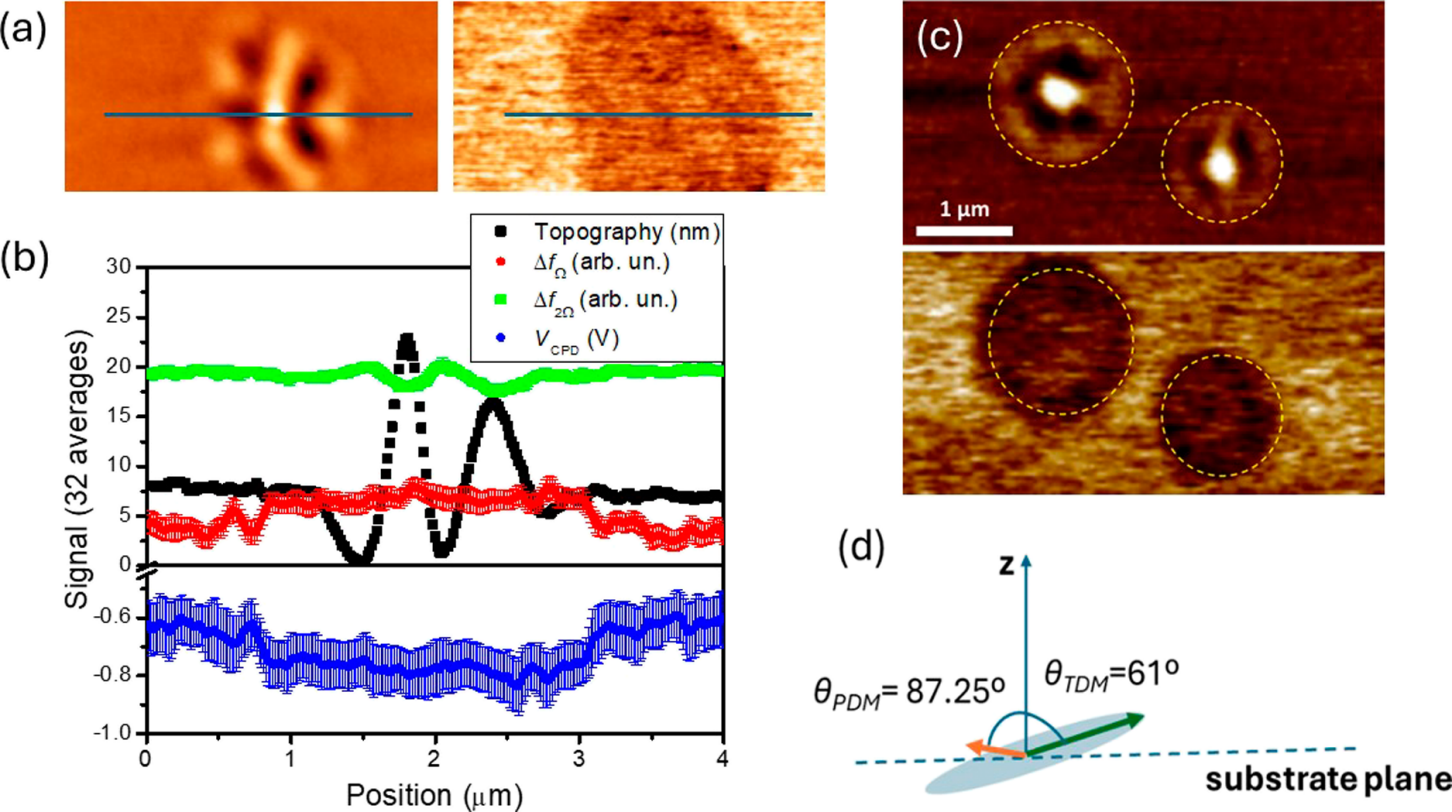
(a) Topographic (left) and Δ*f*_Ω_ (right) images of a 5 μm × 3 μm area containing ultrastable and liquid-cooled regions. (b) Line scans across the line shown at the top images of the 4 scanning modes: Topography (black symbols), Δ*f*_Ω_ (red symbols), Kelvin potential (blue symbols) and Δ*f*_2Ω_ (green symbols). Uncertainties of all signals are reported as error bars. (c) Topography (top) and KPFM (bottom) of two LCG regions. The scale bar is 1 μm and the average surface potential difference between the two regions in this image is around 130 mV. (d) Average relative orientation of the long axis of the molecule and the PDM in the UG with respect to the vertical direction.

From the average value of the relative variation of surface potential of the UG with respect to the LCG and assuming the isotropic liquid-cooled glass shows a negligible surface potential, we can estimate the average orientation of the PDM of the TPD molecules in the UG matrix. The surface potential can be written as^18^

6 $$SP=\frac{pdn\left\langle \cos{\;\theta }_{PDM}\right\rangle }{\varepsilon_{r}\varepsilon_{0}}$$

where *p* is the value of the PDM, *d* the thickness of the film, *n* the number density of molecules, ε_r_,ε_0_ the dielectric constant and the vacuum permittivity, respectively and θ_PDM_ the angle between the permanent dipole moment and the surface normal (Figure 3d). The permanent dipole moment (PDM) of a single TPD molecule, calculated using DFT, is *p* = 0.41. This value is not expected to change significantly within the glass, as our DFT calculations indicate that TPD has relatively low overall polarizability.^46^ Consequently, intermolecular interactions are unlikely to substantially affect the computed single-molecule values. The thickness, *d* = 63 nm is determined through a calibrated quartz crystal monitor (see Materials and Methods) and $n=\frac{\rho N_{A}}{M}$ is estimated from previous measurements of the density of TPD layers grown under similar experimental conditions measured by ellipsometry, ρ = 1.08 g/cm^3^, and molecular weight *M* = 516.6 g/mol, while ε_r_ = 3.5.^51^ Using these numbers, we obtain a value for ⟨cos θ_PDM_⟩ = 0.049 ± 0.006, which accounts for an average orientation of the PDM of 2.75 ± 0.05° off the substrate surface (see Figure 3d). This indicates that a very slight deviation from a random molecular distribution result in a measurable SOP, even in glasses with small PDM as TPD. The schematic in Figure 3d illustrates the relative orientation of the molecule’s long axis and its permanent dipole moment (PDM) with respect to the substrate normal. TPD glasses grown at 0.85 *T*_g_ also exhibit some layering in the *z*-direction, as indicated by wide-angle X-ray diffraction measurements on similar samples (data not shown).^49^ Several previous publications^22,24^ have reported the loss of molecular orientation in vapor-deposited ultrastable glasses upon transformation into liquid-cooled glass using wide-angle X-ray scattering. The combination of UV/vis spectroscopy, EFM measurements, and previous X-ray diffraction studies on ultrastable glasses provides a comprehensive view of this phenomenon upon heating above *T*_g_.

We investigated the temporal evolution of several nuclei during thermal treatments. Specifically, the data in Figure 4 correspond to an as-deposited trilayer that underwent partial transformation within the AFM chamber at *T*_g_ + 12 K (345 K) over 4 h. Subsequently, the temperature was reduced to *T*_g_ + 8 K (341 K), and the nuclei’s evolution was monitored in situ during the thermal treatment. Figure 4b,c show representative images of the nuclei development, captured using both topography and Δ*f*_Ω_ modes. The radial progression of the liquid phase was tracked through more than 60 images acquired over a period exceeding 20 h, as shown in Figure 4a. The radius of the liquid regions was determined by averaging measurements taken along four directions at right angles. As noted earlier, the electrical signal extends slightly beyond the mechanical signature, offering higher spatial resolution of the liquid domains, an advantage over topographic AFM. This enhanced resolution could enable a novel experimental approach to precisely map the geometry of the glass–liquid interface, offering sufficient detail to infer the length scale of dynamic heterogeneities in the liquid, as recently suggested by Herrero et al.^52^

**Figure 4:**
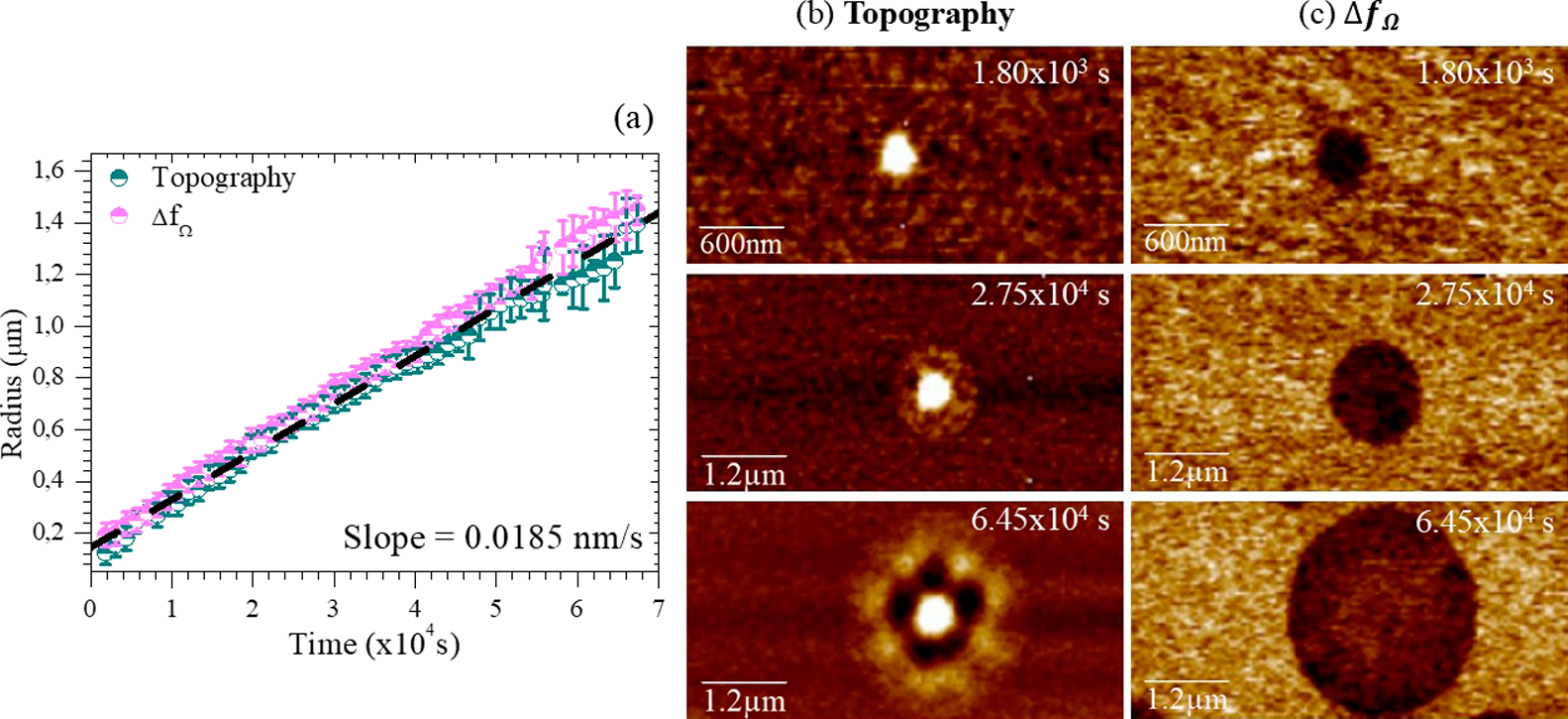
(a) Evolution of radius versus time of a liquid region both in topography (blue/green) and Δ*f*_Ω_ (pink) during a thermal treatment at *T*_g_ + 8 K. The black dashed line represents a linear growth velocity of 0.0185 nm/s. (b) Topography images; (c) simultaneous Δ*f*_Ω_ signal images.

From the onset of detection, the radius grew linearly at a velocity of 0.018 ± 0.002 nm/s (dashed line in Figure 4a), with comparable values for both data sets. The deviation from the linear trend observed in the topography data for larger radius is likely related to the difficulty of establishing clear edge boundaries between transformed and nontransformed regions. Measurements from additional nuclei revealed growth velocities in the range of 0.014–0.018 nm/s, consistent with the growth front velocities observed in uncapped TPD UG layers.^6^ This result is also in agreement with our previous measurements on the transformation of capped films by AFM^13^ where we showed that once the liquid regions start to grow, they do it at a constant rate that fits with the front velocity at that specific temperature.

## Conclusions

We have provided unequivocal evidence of the formation of liquid domains during the devitrification of an ultrastable glass above *T*_g_ by combining topographical imaging with electrostatic force microscopy. The relaxation of anisotropy in vapor-deposited glass into the isotropic configuration of liquid-cooled glass generates a distinct electrical contrast between the two regions. The electrical signal variation associated with these liquid regions surpasses mere topographic contrast, offering a highly sensitive proxy for assessing the thermal stability of organic thin-film glasses. Additionally, we demonstrate that liquid growth, which progressively consumes the glass, occurs via dynamic facilitation and maintains a constant rate from the earliest stages of formation. These findings underscore electrical-based force microscopy as a powerful tool to address key challenges, such as resolving the sharpness of the glass/liquid interface, probing the thermal stability of organic semiconductors, and assessing surface potential retention. The latter has received renewed interest in the organic electronics community due to its crucial influence on device efficiency and operational lifespan.
